# Molecular characterisation of *Giardia duodenalis* from human and companion animal sources in the United Kingdom using an improved *triosephosphate isomerase* molecular marker

**DOI:** 10.1016/j.crpvbd.2022.100105

**Published:** 2022-11-26

**Authors:** Sarah Krumrie, Paul Capewell, Mike McDonald, Dawn Dunbar, Rossella Panarese, Frank Katzer, Noha El Sakka, Dominic Mellor, Claire L. Alexander, William Weir

**Affiliations:** a School of Biodiversity, One Health and Veterinary Medicine, College of Medical, Veterinary and Life Sciences, University of Glasgow, Garscube Estate, 464 Bearsden Road, Glasgow, G61 1QH, UK; b BioClavis Ltd., Queen Elizabeth Teaching and Learning Centre, 1345 Govan Road, Glasgow, G51 4TF, UK; c Moredun Research Institute, Pentlands Science Park, Edinburgh, EH26 0PZ, UK; d Scottish Microbiology Reference Laboratories (Glasgow), New Lister Building, 10-16 Alexandria Parade, Glasgow Royal Infirmary, Glasgow, G31 2ER, UK; e Public Health Scotland, Meridian Court, 5 Cadogan Street, Glasgow, G2 6QE, UK; f National Health Services Grampian, Aberdeen Royal Infirmary, Aberdeen, AB25 2ZN, UK

**Keywords:** *Giardia*, Genotyping, *tpi*, New primers, Zoonotic, Epidemiology, Feline, Canine

## Abstract

*Giardia duodenalis* is a protozoan parasite known for its ability to cause gastrointestinal disease in human and non-human mammals. In the UK, the full impact of this parasite has yet to be fully explored, due to the limited testing which has been undertaken in humans and the low-resolution assemblage-typing methods currently available. Rather than being primarily a travel-associated condition, a recent study has highlighted that an endemic *Giardia* cycle is present in the UK, although the source of human disease is unclear in the majority of cases. This study focussed on the improvement of one of the commonly used assemblage-typing assays, a nested *topoisomerase phosphate* (*tpi*) PCR, to increase the amplification success rate across both human and companion animal samples. After comparing published primers to full *Giardia* reference genomes, this marker protocol was optimised and then deployed to test a substantial number of human (*n* ​= ​79) and companion animal (*n* ​= ​174) samples to gain an insight into the molecular epidemiology of *Giardia* in the UK. One assemblage A1 and eleven assemblage A2 genotypes were detected in humans, along with and 25 assemblage B genotypes. Assemblage A1 genotypes, known to be human-infective, were found in three feline and one canine sample, while one feline sample contained assemblage A2. Additionally, four feline samples contained assemblage B, which is recognised as potentially human-infective. This study demonstrates the presence of potentially human-infective *Giardia* genotypes circulating in the companion animal population, notably with 17.4% (8/46) of feline-derived *Giardia* strains being potentially zoonotic. Using a modified *tpi*-based genotyping assay, this work highlights the potential for domestic pets to be involved in the endemic transmission of giardiasis in the UK and underlines the need for appropriate hygiene measures to be observed when interacting with both symptomatic and asymptomatic animals. It also serves to underline the requirement for further studies to assess the zoonotic risk of *Giardia* associated with companion animals in high-income countries.

## Highlights

• Currently used *Giardia* genotyping markers have poor success rates with companion animal samples.
• A *triosephosphate isomerase* marker was modified and improved upon using genomic sequence data.
• This novel marker assay was applied to genotype a large collection of *Giardia* from humans and companion animals in the UK.
• Potentially human-infective parasite strains were detected in an appreciable proportion of UK cats.
• Importance of companion animals in endemic *Giardia* infection in high-income countries should be further investigated.

## Graphical abstract

**Figure:**
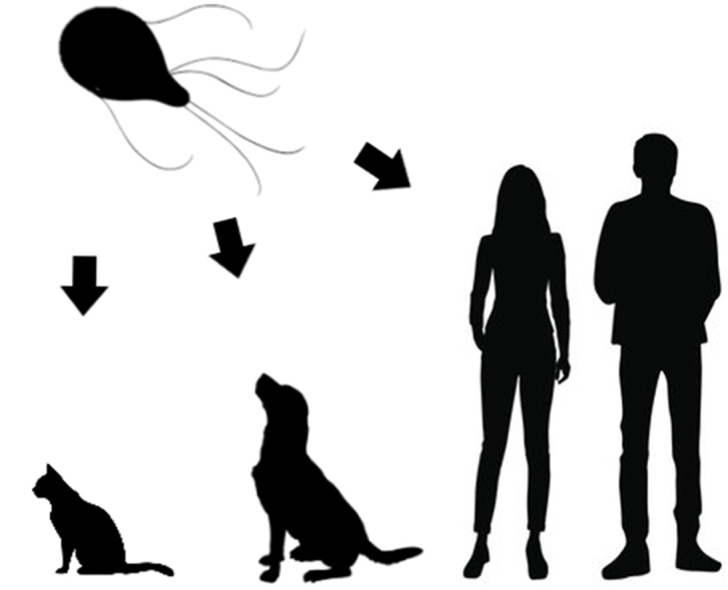

## 1 Introduction

*Giardia duodenalis* (referred to as *Giardia* hereafter) is a binucleate flagellated protozoan found worldwide that infects the gastrointestinal system of a wide variety of mammals, including humans (Adam, 2000, 2001; Caccio & Ryan, 2008). Although it primarily causes diarrhoea, it can also result in serious long-term sequelae. In fact, giardiasis may affect the growth and development of young children in low- and middle-income countries, and a study in India found children infected with *Giardia* consistently showing cognitive deficits compared to others of their age (Simsek et al., 2004; Ajjampur et al., 2011; Jethwa et al., 2015). Additionally, a prolonged chronic colitis can result as a secondary effect of giardiasis (Hanevik et al., 2009, 2014; Wensaas et al., 2012; Dann et al., 2018). Asymptomatic infections also commonly occur (Caccio & Ryan, 2008; Thompson & Ash, 2016).

Humans and animals become infected by ingesting the cystic stage of *Giardia*, which is carried *via* faeces, through a variety of routes. These cysts are highly environmentally resistant and can survive outside the host for days to months, depending on variables such as location, surrounding temperature and amount of organic matter present (Alum et al., 2014). In order to better understand the transmission dynamics between animals and humans, there have been increasing calls for improved methods for genetic characterisation (Caccio et al., 2005; Savioli et al., 2006; Caccio & Ryan, 2008; Durigan et al., 2018). To date, *Giardia* is considered a single species, although multiple genotypically distinct sub-types have been defined. These sub-types are termed “assemblages” and exhibit varying degrees of mammalian host specificity, with some assemblages found in multiple host species (Adam, 2000, 2001; Read et al., 2004; Caccio & Ryan, 2008; Lebbad et al., 2010). Eight assemblages have been defined and assigned with the letters A through to H. Assemblage A is a potentially zoonotic human type which may be divided into sub-assemblages A1, A2 and A3. A1 has the potential to infect humans as well as a range of other mammals whereas A2 is generally found in human hosts, and A3 can be found in wild hoofed animals (Zajaczkowski et al., 2021). Assemblage B also infects humans and other mammals whilst assemblages C and D are considered canine assemblages. Assemblage E is primarily found in livestock, assemblage F in felids, assemblage G in murids and assemblage H in seals and gulls (Heyworth, 2016). The accurate identification of assemblage types can aid in detecting outbreaks, determining or predicting directionality of infection, and in the general epidemiological monitoring of the parasite.

The genes *beta giardin* (*bg*), *glutamate dehydrogenase* (*gdh*) and *triosephosphate isomerase* (*tpi*) were evaluated by Caccio et al. (2002), Read et al. (2004) and Sulaiman et al. (2003), respectively, for their degree of polymorphism in the *Giardia* population, which was found to be sufficient to draw phylogenetic inferences to assemblage and sub-assemblage level. However, genetic markers based on these genes have proven to be inconsistent in terms of PCR success rate and genetic classification, and only limited further development of markers based on *bg* and *tpi* has been undertaken. Thus, many studies have been reliant on the original published markers (Robertson et al., 2007; Volotão et al., 2007; Caccio & Ryan, 2008; Abe et al., 2010; Daly et al., 2010; Lebbad et al., 2010; Yang et al., 2010; Colli et al., 2015; Nolan et al., 2017; Abd El-Latif et al., 2020; El Bakri et al., 2021). Additionally, the need for consistent and reproducible methods of DNA extraction prior to PCR in order to maximise the amount of *Giardia* DNA recovered from samples has been highlighted (Thompson & Ash, 2016), although no consensus has been achieved. If isolates cannot be amplified or definitively placed within a specific assemblage, it limits the capacity of the marker to be applied to outbreak analysis and elucidate transmission pathways (Thompson & Ash, 2016).

Many of the current PCR primers contain several degenerate bases, which increases the possibility of off-target primer binding. While this approach can accommodate a degree of genetic variation within and between assemblages, it means that only a proportion of the primers in any reaction will match the template at the degenerate positions. Additionally, when phylogenetic comparisons are undertaken using each of these genes, conflicting results may arise (Caccio et al., 2008). For example, results of *gdh* typing have shown a discrepancy the 18S genotyping results (Read et al., 2004; Traub et al., 2004). In particular, the *tpi* locus has been hampered by a low amplification success rate and has been highlighted as a good candidate for further development (Zajaczkowski et al., 2021).

*Giardia* causes giardiasis in both low-, middle- and high-income countries. The World Health Organization highlighted giardiasis as a “Neglected Disease” in its 2004 initiative to identify and eliminate diseases directly contributing to human sickness and death (Savioli et al., 2006). In low- and middle-income countries giardiasis is linked to poor sanitation, but in high-income countries human outbreaks of varying size and sporadic cases are often caused by ingestion of contaminated water (Mahbubani et al., 1992; Adam, 2001; Caccio & Ryan, 2008; Daly et al., 2010). Contaminated food or food handlers are also responsible for many *Giardia* outbreaks in humans.

In the UK, an audit of Scottish National Health Service (NHS) Microbiology Laboratories highlighted that diagnostic testing for *Giardia* was mostly only undertaken in individuals when a history of recent travel was reported by a patient, which resulted in under 20% of diarrhoeic samples being screened for the parasite (Alexander et al., 2017; Ferguson et al., 2020). However recently, when a Scottish local health board began testing all submitted faecal samples for *Giardia* using an enzyme immunoassay (EIA), it was discovered the parasite was acquired locally as well as abroad, demonstrating its endemic status in Scotland (Currie et al., 2017; Ferguson et al., 2020). A case-control study in England also found *Giardia* infection to have a local transmission route in addition to travel, which included a significant correlation between owning a dog and harbouring an assemblage A infection (Minetti et al., 2015). This study did not extend to detecting or genotyping *Giardia* from animals related to the human giardiasis cases, unfortunately. While companion animals have been screened for zoonotic assemblages on all continents and livestock have been similarly tested in France, Germany, Italy and the UK (Sprong et al., 2009; Geurden et al., 2012; Bartley et al., 2019; Horton et al., 2019), the assemblage data for companion animals in the UK, and Scotland in particular, is unclear apart from one canine assemblage A specifically located in a London canine shelter (Upjohn et al., 2010; Feng & Xiao, 2011).

The first objective of the present study was to utilise published genomic sequence data to refine the *tpi* marker in order to improve sensitivity, in terms of its ability to detect a range of genotypes when applied to a panel of *Giardia* qPCR-positive field samples. In the course of this endeavour, an optimal method for DNA extraction from *Giardia* cysts in faecal material was also determined.

The second objective of the study was to utilise the improved marker to characterise a national collection of companion animal and human samples to evaluate host specificity of *G. duodenalis* in a high-income country, i.e. the UK. In addition to experimentally validating the markers, it was hoped this would provide new insights into host specificity of assemblages in the context of a high-income country.

## 2 Materials and methods

### 2.1 Parasite material

Faecal samples from companion animals were obtained from the University of Glasgow’s Veterinary Diagnostic Services (VDS). This comprised samples sent to the laboratory to investigate infectious causes of diarrhoea in animals attending a variety of veterinary clinics primarily in the UK between January 2018 and June 2021. DNA extracts of companion animal faecal samples, which were found to be *Giardia-*positive by a diagnostic RT-qPCR (Verweij et al., 2003), were retained for the present study and were stored at −80 ​°C. A total of 174 feline and canine samples with Ct-values ranging from 17 to 39 were collected.

Human faecal samples containing *Giardia* were obtained from the national Reference Laboratory collection within the Scottish Microbiology Reference Laboratories, Glasgow (SMiRL) which forms part of the National Health Service (NHS) in Scotland. Surplus samples were tested *via Giardia lamblia* antigen-based EIA (Catalogue number GL2-96, Launch Diagnostics, Kent, UK) that had been submitted for routine parasite investigations, therefore no additional samples were requested. Samples were fully anonymised, and no patient identifiers were released to protect patient confidentiality. A total of 79 human faecal samples submitted from Scottish cases between September 2019 and March 2020 were collected and stored in Faeces Stabilisation Buffer (Stratec, Birkenfeld, Germany) at −4 ​°C before DNA extraction for the present study.

Seven known positive DNA extracts were selected from VDS stock to optimise the newly designed primers. Four canine and three feline samples with Ct-values ranging from 17 to 33 were selected to span the range of Ct-values and represent the main species for which samples are processed by the VDS.

### 2.2 DNA extraction

A preliminary comparison of extraction methods was performed using 0.2 ​g sub-samples of companion animal faecal material. Human faecal material was reserved for use with the final optimised primers as there was limited material per sample and so it was used sparingly. Using the standard manufacturer-recommended protocols, results were compared for (a) the taco™ Nucleic Acid Automatic Extraction System (GeneReach, Taichung City, Taiwan); (b) repeated freeze-thaw using liquid nitrogen followed by a PSP stool kit (Stratec, Birkenfeld, Germany); and (c) bead beating with a Tissuelyser (Qiagen, Hilden, Germany) followed by a PSP stool kit. A comparison of Ct-values from a *Giardia*-specific RT-qPCR on the various extracts indicated that the taco™ method generated the highest concentration of recovered *Giardia* DNA and it was therefore selected for use in this study. Thus, for each companion animal sample, approximately 0.2 ​g of faecal material was placed into an Eppendorf tube containing 1 ​ml of lysis buffer containing polyvinylpolypyrrolidone (PVPP). Lysis buffer consisted of Triton X-100, poly(vinylpolypyrrolidone), diaminoethanetetra-acetic acid disodium salt dihydrate and guanidine thiocyanate. Next, 100 ​μl of a solution containing a known quantity of feline herpesvirus (FHV) was added to act as extraction control for the RT-qPCR reaction. The tube was vortexed and left to sit for 10 ​min, with another brief vortex after 5 ​min to obtain a homogenous solution. The sample was then centrifuged for 10 ​min at 15.7× *g*. The taco™ Nucleic Acid Automatic Extraction System was then employed, which utilised magnetic bead separation technology. The left-most well of each taco™ plate was loaded with 200 ​μl of PVPP-mix supernatant, which were pre-loaded by the manufacturer and contained lysis buffer and magnetic beads. Each plate contained 48 pre-loaded wells and was used for the simultaneous extraction of 8 samples. The wells contained a series of wash buffers, with the final well containing a proprietary elution buffer. This plate was loaded into a taco™ machine, which performed a dual DNA/RNA extraction cycle over the course of 30 ​min. If a sample was to be utilised for Sanger sequencing following PCR analysis, the buffer in the final cell of the plate was replaced with 200 ​μl of dH_2_O. DNA extracts were then removed from the right-most well and placed into new Eppendorf tubes to be stored at −80 ​°C until further analysis was to take place. Human samples were extracted using a PSP Spin Stool DNA Plus Kit (Stratec, Birkenfeld, Germany) according to the manufacturer’s instructions following three rounds of freeze-thaw in liquid nitrogen. A different extraction method was used for these samples as there was no access to the taco™ machine in the human ethics-approved laboratory where they were extracted.

### 2.3 Designing and optimising PCR primers

The five whole-genome sequences currently available in GiardiaDB (https://giardiadb.org/), representing assemblages A, B and E, were queried and five *tpi* gene sequences were downloaded as FASTA files together with 1 ​kb of upstream and downstream sequence data. The sequences were then aligned and trimmed to the published *tpi* primer sites (Sulaiman et al., 2003) using Geneious Prime (Dotmatics). The published primers for *tpi* were directly compared with the corresponding genomic loci using ClustalX2 (Larkin et al., 2007). New primers were designed by modifying the existing primers to match bases in the full genomic alignment. New *tpi* primers were ordered from Eurofins Genomics (Ebersberg, Germany) and temperature gradients and concentration grids performed on 4 samples of canine origin and 3 of feline origin with Ct-values ranging from 17 to 33, as determined by the VDS diagnostic RT-qPCR upon intake (Verweij et al., 2003), to determine the optimal PCR conditions. A *Giardia*-rich positive control DNA sample representing assemblage A1 (genome WB clone 6) derived from sterile, laboratory-cultivated trophozoites at a diluted concentration of 1:200 was used as template in the first round PCR. To determine the optimal annealing temperature for both rounds, an annealing gradient of 53.2–66.7 ​°C was assessed first with the internal primers and subsequently with the external primers, with a constant 1 ​pmol concentration of forward and reverse primers used throughout. Following annealing temperature selection, primer concentrations were optimised where the forward and reverse primers of both the internal and the external reactions were tested using all combinations of primers at concentrations of 4 ​pmol, 2 ​pmol, 1 ​pmol and 0.5 ​pmol in a 20 ​μl total reaction volume. Both annealing temperature and primer concentrations for both assays were fully optimised prior to use with field samples.

### 2.4 Improved *tpi* marker PCR conditions

As the original *tpi* PCR assay was developed as a nested protocol, the same approach was applied in the present work. A Qiagen HotStarTaq Plus PCR kit (Qiagen, Hilden, Germany) was used to perform both PCR rounds, using kit-supplied reagents unless otherwise stated. Each assay utilised a 20 ​μl total reaction volume consisting of 10x buffer and 0.1 ​μl of HotStarTaq Plus *Taq*, with the dNTP concentration increased to a final concentration of 200 ​μM by adding 0.16 ​μl of ThermoFisher 25 ​mM dNTP mix (ThermoFisher, Paisley, UK). The first-round assay reaction mix additionally contained 0.5 pM of forward primer and 2 pM of reverse primer while the second round used 0.5 pM of both forward and reverse primers. The first round involved an initial denaturation step of 5 ​min at 95 ​°C followed by 40 cycles of 94 ​°C, 54.2 ​°C and 72 ​°C for 1 ​min each, ending with 10 ​min at 72 ​°C. The external PCR product was diluted 1:1000 in molecular grade dH_2_O before being added to the reaction mix of the second round PCR, which comprised 5 ​min at 95 ​°C followed by 40 cycles of 94 ​°C, 57.8 ​°C and 72 ​°C for 1 ​min each, ending with 10 ​min at 72 ​°C (Table 1).

**Table 1:** PCR conditions which differed between the published protocol by Sulaiman et al. (2003) and the modified protocol.

Primer name	Primer sequence (5′-3′)	Product length (bp)	Buffer^a^	No. of cycles	Annealing T (°C)	Total reaction volume (μl)	Template DNA per reaction (μl)	dNTP (μM)^a^	MgCl_2_ (mM)^a^	*Taq* (U) per reaction	Primer (nM)^a^
AL3543 (Outer forward)	AAATIATGCCTGCTCGTCG	605	1x	35	50.0	100	0.25–2.00	200 each	3	5	200
AL3546 (Outer reverse)	CAAACCTTITCCGCAAACC										200
AL3544 (Inner forward)	CCCTTCATCGGIGGTAACTT	530	1x	35	50.0	100	2.50	200 each	3	5	200
AL3545 (Inner reverse)	GTGGCCACCACICCCGTGCC										200
AL3543Mod (Outer forward)^b^	AAATYATGCCTGCTCGTCG	605	10x	40	54.2	20	3.00	50 each	15 (incorporated into buffer)	0.5	500
AL3546Mod (Outer reverse)^b^	TGGCCACCACRCCCGTGCC										2000
AL3544Mod (Inner forward)^b^	CAAACCTTYTCYGCAAACC	531	10x	40	57.8	20	3 ​at 1:1000 dilution of first round PCR product	50 each	15 (incorporated into buffer)	0.5	500
AL3545Mod (Inner reverse)^b^	CCCTTCATCGGYGGTAACTT										500

*Abbreviation*: T, temperature.

a Concentration.

b New primers.

Once the second round of the PCR was completed, 15 ​μl of the product mixed with 3 ​μl of loading dye was subjected to electrophoresis on a 1% agarose gel at 100 ​V for 45 ​min and visualised under UV illumination. The target sequence was predicted to be 531 bp, and any band appearing around this location was excised and extracted using a Qiagen gel purification kit following the manufacturer’s instructions and sent externally for Sanger sequencing (Eurofins). Representative sequences were submitted to the GenBank database under the accession numbers OP860417-OP860514, with assemblage information in the uploaded sequence name and notes.

### 2.5 Amplicon cloning and sequencing

A PCR pJET cloning kit (ThermoFisher Scientific, Paisley, UK) was used per the manufacturer’s instructions to clone and amplify low concentration amplicons from the second round PCR. pJET plasmids were purified from 5 ​ml of Luria Broth (LB) overnight culture using a Qiagen miniprep kit (Qiagen). Three colonies were picked for each isolate and each eluted into 50 ​μl water. The samples were analysed on a Qubit 4 (ThermoFisher Scientific) to confirm there was at least 25 ​ng of plasmid DNA for a 500 bp target product, which was then diluted to 5 ​ng/μl for Sanger sequencing using a Mix2seq kit (Eurofins). Two tubes of 15 ​μl extract were sent to Eurofins for sequencing, containing either 2 ​μl of the forward or reverse primer at 10 ​pmol/μl.

### 2.6 Phylogenetic and statistical analysis

Amplicon sequences along with reference genomes WB-A1, DH-A2, GS-B, KT728520_C, P15-E and KP866788_F for the *tpi* sequence were aligned using CLUSTAL Omega (Sievers et al., 2011), trimmed to the same length and maximum likelihood phylogeny estimated using RAxML (Stamatakis, 2014) with 100 bootstrap iterations. Resulting Newick trees were visualised using FigTree (Rambaut, 2014). Diagnostic assay Ct-values of amplifying and non-amplifying samples (with respect to the new *tpi* protocol) were compared using the Wilcoxon Rank Sum test.

## 3 Results

### 3.1 Primer analysis and redesign

**Fig. 1:**
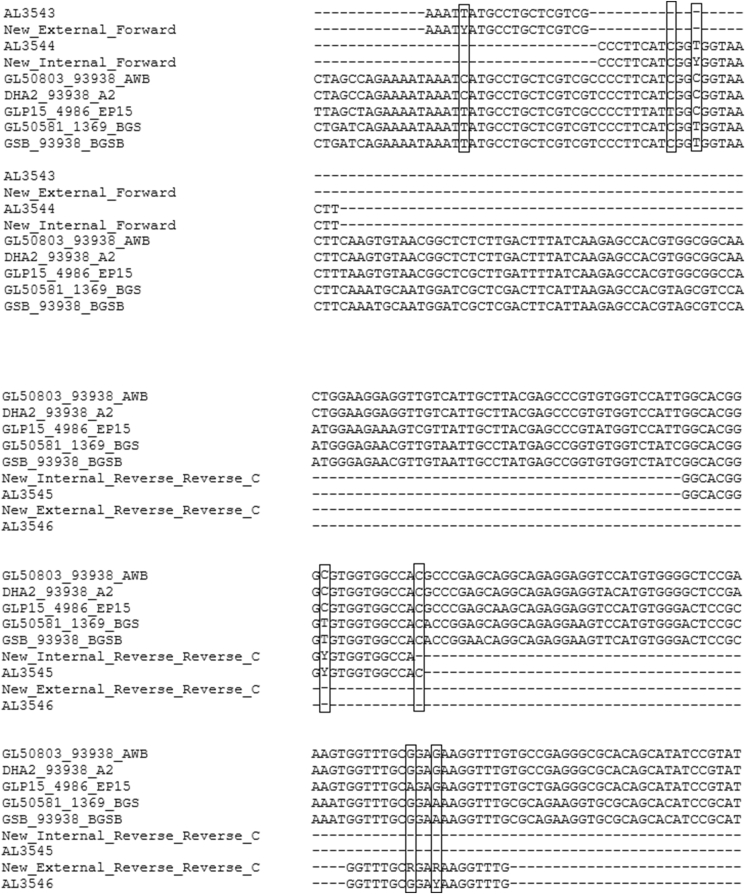
Alignment of five full *Giardia* genomes along with published and modified primers with differences outlined by a box. The vertical line with two short angular lines represents a gap in the sequence alignments to highlight the primers. GL50803_93938_AWB: Assemblage A1; DHA2_93938_A2: Assemblage A2; GLP15_4986_EP15: Assemblage E; GL50591_1369_BGS: Assemblage B; GSB_93938_BGSB: Assemblage B.

The published primer sequences were compared with available complete *Giardia* genomic sequences at the *tpi* locus to detect mismatches in the primer sequences that may explain PCR failure (Fig. 1). In total, six mismatching positions were identified between the published primers and the full genomes. These bases were either replaced with appropriate degenerate bases or the primer was shifted slightly. Care was taken to avoid hairpin formation, provide optimal GC content and match annealing temperatures (Northwestern University’s OligoCalc: Oligonucleotide Properties Calculator). For one primer, the internal forward, one base was not amended as the replacement base would have provided an unsuitable melting temperature (under 50 ​°C) and the primer could not be shifted without significantly altering melting temperature. A single base was also removed from the end of the internal reverse primer to bring the melting temperature from 68–70 ​°C to 66–68 ​°C. The newly designed primers for first round (external) amplification were 5′-AAA TYA TGC CTG CTC GTC G-3′ (Forward) and 5′-CAA ACC TTY TCY GCA AAC C-3′ (Reverse). The suggested names for these primers are AL3543Mod and AL3546Mod, respectively. The primers used for the internal second round were 5′-CCC TTC ATC GGY GGT AAC TT-3′ (Forward) and 5′-TGG CCA CCA CRC CCG TGC C-3′ (Reverse), with the suggested names AL3544Mod and AL3545Mod respectively.

### 3.2 Optimisation of PCR conditions for the new primers

**Fig. 2:**
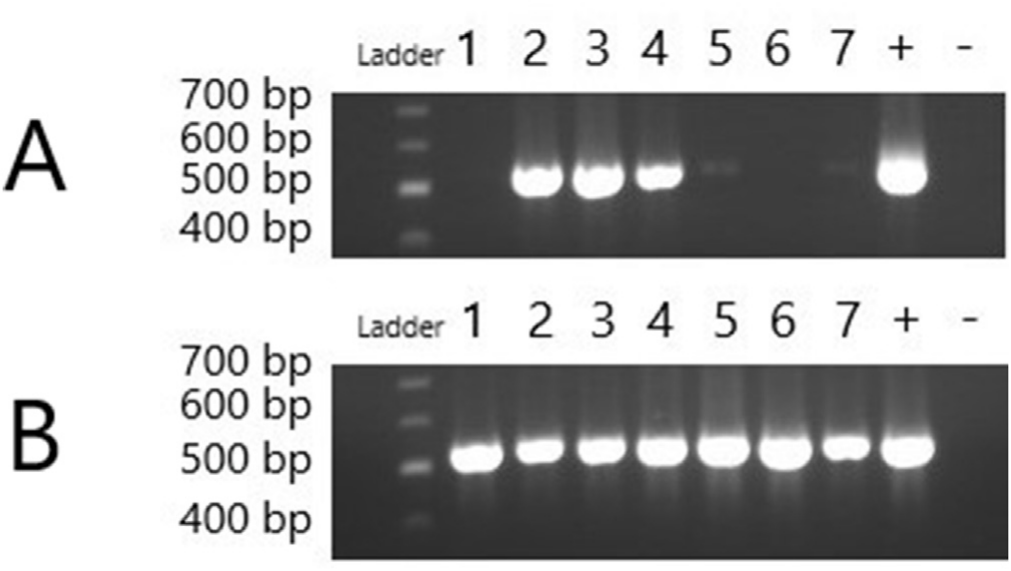
PCR amplification of panel of seven samples: target product at 531 bp. **A** Published *tpi* primers using published conditions on seven field samples from Veterinary Diagnostic Services. **B** Modified *tpi* primers using optimised conditions on the same seven field samples.

The newly designed primers were tested to find the optimal annealing temperature and oligo concentration for each round of the nested PCR assay. For the first-round external primers, the optimal annealing temperature was found to be 54.2 ​°C, with concentrations of 0.5 pM and 2 pM for forward and reverse primers, respectively. The second-round internal primers were found to have an optimum annealing temperature of 57.8 ​°C with concentrations of 0.5 pM each for forward and reverse primers. Following a series of test dilutions of 1:100, 1:500 and 1:1000, the optimal dilution of the primary product for the second-round reaction was found to be 1:1000. Following optimisation, both sets of markers were tested on 7 field samples picked to represent a range of parasite loads, as inferred from diagnostic RT-qPCR Ct-values, and host species together with the DNA extract of the sterile trophozoites as a positive control. These 7 samples were used with the initial PCR primers at the beginning of the project and throughout the troubleshooting process, then retested with the modified primers at the end of the designing and optimising phase (Fig. 2). The published assay was able to generate clear, convincing bands from 3 samples together with weak amplicons from a further 2, which were insufficient for Sanger sequencing. In contrast, the novel assay was able to generate strong bands from each of the 7 DNA samples. These samples were sequenced and found to represent assemblages A, C and F.

### 3.3 Genotyping of Scottish human- and animal-derived *Giardia*

**Fig. 3:**
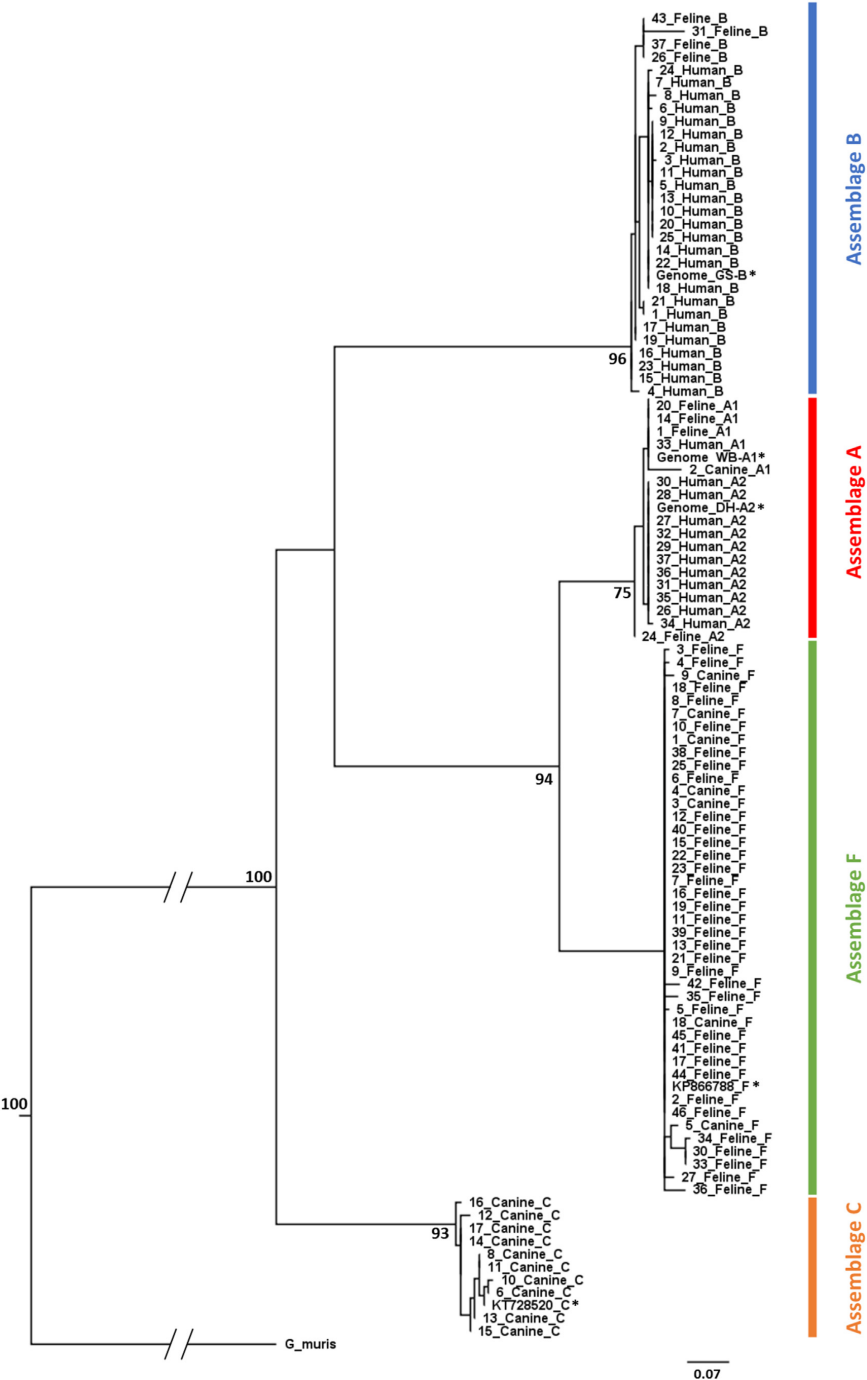
Phylogenetic tree representing the assemblage distribution of the field sample amplicons generated. Bootstrap values on major branches are included. The scale of the genetic distance is indicated. ∗ indicates reference genome. Data are available on the GenBank database under accession numbers OP860417-OP860514.

The redesigned and optimised *tpi* PCR assay was applied to 174 companion animal and 79 human faecal samples positive for *Giardia* by RT-qPCR and *G. lamblia* antigen-based EIA, respectively. Of these samples, 73 companion animal samples (41.95%, 73/174) and 37 human samples (46.84%, 37/79) resulted in bands being generated at the expected location and these were sent for Sanger sequencing. Unfortunately, 8 companion animal samples did not produce bands of sufficient concentration to sequence, which led to sequences being generated for a total of 65 companion animal and 37 human sample amplicons. This corresponded to genotyping success rates of 37.36% and 46.84%, respectively. Using 99 samples for which full-length good-quality sequence was obtained, a cladogram was constructed incorporating published sequences representing assemblages A1, A2, B, C and F trimmed to the *tpi* locus with *Giardia muris* as an outgroup (Fig. 3). Two assemblage F sequences were excluded due to insufficient length. The field samples formed four discrete clusters containing assemblage A, B, C and F reference sequences. The sequences clustered into monophyletic groups and while there was strong bootstrap support for most major branches of the tree, there was some ambiguity as to the relative position of clades B, C and A/F. The tree supports the current delineation of *G. duodenalis* into assemblages *via* the representative sequences of assemblages A, B, C and F, revealing that the amplicons generated in this study represented a mixture of these four assemblages.

A summary of the genotyping results is shown in Table 2. Human samples generated twelve assemblage A (one A1 and eleven A2) and 25 assemblage B amplicons, with these two human-associated assemblages being anticipated in these samples. Four feline and one canine sample were found to contain assemblage A, while four feline samples contained assemblage B. Three of the four feline samples and the canine sample categorised as A were sub-typed as A1 and the remaining feline sample was sub-typed as A2. The remainder of the canine samples, anticipated to correspond to either of the putatively canine-specific assemblage C or D, was found to be a mixture of assemblages C and F. Other than the four B assemblages, amplicons derived from feline samples, corresponded with the putatively feline-specific assemblage F, as expected. Eight companion animal samples produced faint bands which were too weak to sequence. The Ct-values of the companion animal samples which could be typed ranged from 17 to 37, while those that could not, ranged from 23 to 36. Overall, the Ct-values of the non-*tpi* amplifying samples were found to be significantly higher (Wilcoxon Test, *P* ​≤ ​0.001), indicating that parasite DNA concentration within samples is a contributing factor explaining the success or failure of the novel PCR and consequently the ability to genotype samples.

**Table 2:** A summary of human and companion animal samples tested using the modified *tpi* primers which had sufficient DNA concentration for Sanger sequencing.

Assemblage	Human samples (*n*/*N*)	Canine samples (*n*/*N*)	Feline samples (*n*/*N*)
A	12/79	1/52	4/122
	(A1: *n* ​= ​1; A2: *n* ​= ​11)	(A1: *n* ​= ​1; A2: *n* ​= ​0)	(A1: *n* ​= ​3; A2: *n* ​= ​1)
B	25/79	0/52	4/122
C	0/79	10/52	0/122
F	0/79	8/52	38/122
Total genotyped samples/species	37/79	19/52	46/122

*Abbreviations*: *n*, no. of samples successfully sequenced; *N*, no. of samples tested.

## 4 Discussion

The modified *tpi*-based genotyping assay described here may be used to classify human- and animal-derived isolates of *Giardia* into the currently accepted assemblages with improved sensitivity, in terms of the proportion of samples that can be successfully genotyped (Wang et al., 2017; Rafiei et al., 2020; Zajaczkowski et al., 2021). However, the failure to amplify from a marked proportion of field samples reflects the ongoing challenge of genotyping *Giardia* isolates. This is an issue shared with markers based on *bg* and *gdh* loci unless the assemblages of interest are limited to A and B, which appear to be associated with a higher success rate (Correa et al., 2020; Rafiei et al., 2020; Zajaczkowski et al., 2021; Calegar et al., 2022). The tendency of *tpi* to be used in studies where animal samples are involved assisted in the decision to focus on improving this particular marker (Caccio et al., 2008; Lebbad et al., 2010; Zou et al., 2021). We demonstrate that this new assay can amplify a wider range of field samples than the published protocol, as illustrated in Fig. 2. The higher level of success with the new primers illustrates that amplification failure associated with the existing primers may be explained, in some cases, by hitherto unappreciated polymorphism at the primer binding site, which would cause a mismatch in bases and prevent PCR cycling.

The relatively recent trend in publishing *Giardia* genotyping PCR amplification success rates allows the comparison of these modified primers to the original published primers. The amplification rate of companion animal and human samples was 73/174 (41.95%) and 37/79 (46.8%), respectively. When compared with the success rates of *tpi* quoted in similar studies, the companion animal success rate of *tpi* in this study was consistently much higher, while the human success rate was either higher or lower, depending on the study (Rehbein et al., 2019; Sommer et al., 2018; Correa et al., 2020; Zajaczkowski et al., 2021; Wu et al., 2022). In the present study, samples with a higher parasite load were associated with a higher likelihood of genotyping success. This is logical, and an even stronger correlation may have been observed if the primers had been able to capture more of the allelic polymorphism suspected to exist in the *Giardia* population. It is possible the greater amount of parasite genetic material in samples with lower diagnostic PCR Ct-values increases the likelihood of partial or imperfect binding, sufficient to initiate a PCR reaction. However, the failure of samples with relatively low Ct-values (as low as 23), indicating substantial parasite load, suggests there is likely further undocumented primer-site polymorphism preventing amplification. Genetic polymorphism in the parasite population appears to be a major factor in determining the success or failure of PCR-based genotyping methods and further investigation into other genetically informative loci is warranted to underpin development of an effective multi-locus genotyping approach. By integrating new information from the ever-expanding number of genomic sequences into the improvement of *Giardia* genetic markers, it may be predicted that the rate of genotyping success will increase together with confidence in assigning assemblages. It is possible that the amount of diversity in the field population of *Giardia* is sufficiently great that, even at single genetic loci, several assemblage-specific PCRs may be required, which could be developed as a multiplex PCR.

While many of the *Giardia* assemblages detected in this study were of the anticipated type given their host, some unexpected results were generated. Several canine samples contained assemblage F genotypes; this has not been documented previously, although assemblage F has been found in cetaceans and pigs (Heyworth, 2016). While this may represent true cross-species infection, one may speculate that it could be explained by dogs ingesting *Giardia*-positive feline faecal material and experiencing a transient infection or acting as a transport host, without active infection. Judging by the Ct-range of these samples, which ranged from 24 to 37, either of these scenarios may be possible. This result does, however, call into question how strictly host-specific this and potentially other assemblages truly are. In 2016, Heyworth published a paper detailing the various mammalian hosts associated with each assemblage. The only assemblage that remained strictly within its supposed host niche was assemblage G, which until that point had only been found in rodents (Heyworth, 2016). The findings of the present study and others (Foronda et al., 2008; Cardona et al., 2011; Qi et al., 2015; Caccio et al., 2018; Deng et al., 2018) suggest that the idea of host-specificity should perhaps be better considered as host-propensity. As a greater number of samples from different hosts are typed, the likelihood of finding other assemblages can be better quantified in geographical areas with varied epidemiological situations. In line with these ideals, four feline-derived samples were found to contain classic human assemblages in the present study; these comprised three samples with assemblage A and one with assemblage B, echoing the findings of previous work in low- and high-income countries (Adam, 2000, 2001; Read et al., 2004; Caccio & Ryan, 2008; Lebbad et al., 2010). Assemblage C was detected in a range of canine samples in the present study. In terms of the efficacy of the novel *tpi* assay, this is an encouraging finding as no assemblage C genome was available for the genomic analysis.

While the putative dog- and cat-specific genotypes were not identified in humans, the finding of human-infective assemblages A and B in multiple companion animals in the UK, specifically cats, raises the possibility of zoonotic disease transmission in the domestic setting. This finding goes some way to explaining the results of the previous modelling work which showed dog ownership to be a risk factor for human giardiasis in the UK (Minetti et al., 2015). These findings highlight the importance of observing suitable hygiene measures when handling diarrhoeic companion animals suspected of or diagnosed with *Giardia* infection even in high-income countries, although this will only address symptomatic spread. Asymptomatic carriage and control would require blanket testing of healthy animals, and the same hygiene measures would undoubtedly aid the cessation of further parasitic infection. Heyworth’s study (Heyworth, 2016) cites that assemblages A and B have been found in companion animals in several countries, which further supports the need for appropriate biosecurity measures around diarrhoeic animals or where companion animals may be around eating surfaces such as in cat cafes (Lebbad et al., 2010; Covacin et al., 2011; Suzuki et al., 2011; Volotão et al., 2011; Li et al., 2013).

The development of higher resolution markers will allow directionality and origin of infection to be inferred more easily, which would allow public health bodies to develop biosecurity measures specific to the major sources of infection in their region. In high-income countries, this may mean paying particular attention to companion animals and water purification (Krumrie et al., 2022). While attention is classically paid to contaminated water in low-income countries (Squire & Ryan, 2017; Aw et al., 2019; Saaed & Ongerth, 2019), the availability of knowledge of companion animal infection may also influence biosecurity advice surrounding free-roaming animals to include washing hands whenever contact is made with the animal or with soil in which animals are known to defaecate.

The present study has highlighted not only the possibility of unexpected host-assemblage relationships, but also the presence of zoonotic assemblages in UK companion animal samples. These findings are important to appreciate assemblage types in terms of differing host propensity rather than absolute species-specificity and it would be advantageous if epidemiological models were to incorporate this concept. Additionally, increasing the resolution of markers in terms of sub-assemblage typing would help appreciate and quantify the zoonotic risk that particular strains pose. With more refined genetic markers, medical and public health officials may discover that the actual risk of zoonotic infection may be higher than estimated and more detailed, evidence-based public health advice may be generated to limit parasite spread.

## 5 Conclusions

This study modified a *tpi*-based genotyping assay, accounting, so far as possible, for genetic variation in the parasite genome based on published sequence data and found several human-infectious *Giardia* assemblages in companion animals in a high-income country. This provides further evidence of the zoonotic potential of *Giardia* circulating in domestic dogs and cats and reinforces the need for the pet-owning public to observe appropriate hygiene measures. This work also serves as a basis for further research into potential non-human reservoirs of infection and highlights the lack of strength supporting the assemblage model to which *Giardia* is confined, as unexpected host-assemblage results continue to be discovered. As more comprehensive genotyping methodology is developed, the veracity of the current assemblage paradigm can be reviewed and revised if necessary. With more complete genomic information and refined detection techniques, the molecular epidemiology of *Giardia* can be explored in different areas of the world to aid in determining its significance as a zoonotic pathogen in high-income countries.

## Funding

This study was funded by an award by the 10.13039/501100000589Chief Scientist Office, reference TCS/18/22 and supported by funding from the Scottish Government’s 10.13039/100011310Rural and Environment Science and Analytical Services Division (RESAS).

## Ethical approval

The study was approved by the Ethics and Welfare Committee of the University of Glasgow School of Veterinary Medicine (Ref. EA03/21).

## CRediT author statement

Sarah Krumrie: methodology, software, validation, formal analysis, investigation, data curation, writing - original draft, writing - review & editing, visualisation. Paul Capewell: conceptualisation, methodology, software, validation, formal analysis, investigation, resources, data curation, writing - review & editing, visualisation, supervision, project administration, funding acquisition. Mike McDonald: methodology, validation, resources, writing - review & editing, visualisation. Dawn Dunbar: methodology, software, validation, resources, writing - review & editing, visualisation. Rossella Panarese: validation, investigation, data curation, writing - review & editing, visualisation. Frank Katzer: resources, writing - review & editing, visualisation, funding acquisition. Noha El Sakka: resources, writing - review & editing visualisation. Dominic Mellor: writing - review & editing, visualisation, supervision, funding acquisition. Claire L. Alexander: resources, writing - review & editing, visualisation, funding acquisition. William Weir: conceptualisation, methodology, formal analysis, resources, writing - review & editing, visualisation, supervision, project administration, funding acquisition.

## Declaration of competing interests

The authors declare that they have no known competing financial interests or personal relationships that could have appeared to influence the work reported in this paper. Given their role as Co-Editor, Frank Katzer had no involvement in the peer-review of this article and has no access to information regarding its peer-review. Full responsibility for the editorial process for this article was delegated to Editor-in-Chief Aneta Kostadinova.

## Data Availability

The data supporting the conclusions of this article are included within the article. Representative sequences were submitted to the GenBank database under the accession numbers OP860417-OP860514, with assemblage information in the uploaded sequence name and notes.
